# What Is the Difference Between Laboratory-Scale and Pilot-Scale Grape-Based Products for Older Adults with Chewing Difficulties?

**DOI:** 10.3390/foods13233844

**Published:** 2024-11-28

**Authors:** Ye-Jun Kim, Ji-Hye Ryu, Jin-Young Lee, Yong-Suk Kim, Dasol Kim, Yongseok Kwon

**Affiliations:** 1 Food and Nutrition Division, Department of Agrofood Resources, National Institute of Agricultural Sciences, 166 Nongsaengmyeong-ro, Wanju-gun 55365, Jeollabuk-do, Republic of Korea; backyul99@korea.kr (Y.-J.K.); jhryu13@korea.kr (J.-H.R.); cherry78@korea.kr (J.-Y.L.); 2 Department of Food Science & Technology, Jeonbuk National University, Baekje-daero, Deokjin-gu, Jeonju-si 54896, Jeollabuk-do, Republic of Korea; kimys08@jbnu.ac.kr

**Keywords:** chewing difficulty, grape-based product, lab scale, pilot scale, older adult

## Abstract

This study was conducted to develop a grape product that can facilitate fruit intake for elderly people with chewing difficulties. In addition, the possibility of field application for future prototype production was investigated by comparing laboratory-scale (lab-scale) and pilot-scale production. The stages (stage 1: able to eat with teeth, stage 2: able to eat with gums, stage 3: able to eat with tongue) of the products were determined according to the Korean Industrial Standards for Seniors Friendly Foods (KS H 4897), and the physicochemical composition was measured according to the general test method of the Food Code. The results of this study showed that when comparing the lab scale and pilot scale in stage 1, the hardness of the pilot scale was significantly increased (*p* < 0.05). Conversely, both hardness in stages 2 and 3 and viscosity in stage 3 showed a significant decrease in the pilot scale compared to the lab scale (*p* < 0.05). In addition, pH and sugar acidity were significantly different between the two scales in all stages (*p* < 0.05). These results confirmed the feasibility of developing customized grape products for the elderly with chewing difficulties, and the differences in physicochemical properties between lab-scale and pilot-scale production confirmed the importance of maintaining product quality during scaled-up production. These results can serve as a basis for developing foods for the elderly that require continuous development, and are expected to contribute to improving the dietary habits and quality of life of the elderly with chewing difficulties.

## 1. Introduction

Along with economic growth, life expectancy is increasing due to advances in medicine, improved living standards, and improved living environments, and the elderly population is rapidly increasing worldwide [1,2]. In addition, Korea is one of the countries with the fastest-growing elderly population due to a sharp decline in birth rates and an increase in life expectancy [3]. It is expected that by 2025, we will enter a super-aging society, in which the elderly population (aged 65 or older) will exceed 20% of the total population [4]. Furthermore, Statistics Korea’s Population Projection (2022–2072) predicts that the proportion of the world’s elderly population will increase by 30.3% from 17.4% in 2022 to 47.7% in 2072 [5]. It is believed that government-level management regarding the lives of the elderly will be necessary. Furthermore, The Korea Health Statistics 2022 report shows that seniors aged 65 years or older had an average of 18.5 remaining teeth, with 26.8% of those aged 60 to 69 years and 39.2% of those aged 70 years or older having limited oral function [6]. In addition, 26.4% of those aged 60 to 69 years and 37.9% of those aged 70 years or older reported discomfort when chewing food. The oral health status of an elderly person is determined by the number of remaining teeth, and tooth loss with age weakens their chewing ability [7]. Reduced food intake and digestive problems due to inadequate chewing can lead to nutritional deficiencies, thereby reducing their quality of life [8,9]. The World Cancer Research Fund (WCRF) and the World Health Organization (WHO) recommend a daily intake of more than 400 g of plant foods, including fruits and non-starch vegetables [10,11]. However, elderly people with chewing difficulties in Korea reported consuming only 334.8 g, which is 65.2 g less than the recommended amount [12]. To address this issue, it is necessary to establish intake standards that allow elderly people with weakened or difficult chewing ability to supplement deficient foods, such as fruits and vegetables.

Fruit consumption is essential for obtaining vitamins, minerals, and moisture. It is a rich source of fiber and has antioxidant and anti-inflammatory properties. Consistent and adequate fruit consumption is known to have a positive effect on the prevention and treatment of stroke, cancer, and cardiovascular disease and to reduce mortality [13,14,15]. Therefore, the 2020 Dietary Reference Intakes (KDRIs) for Koreans recommend eating raw fruit once or twice a day [16]. However, as of 2024, only a very small number of the 175 types of exemplary senior-friendly products used fruit, including puree and jelly, and most of them are used in porridge, soft side dishes, or protein supplements [17]. Therefore, it is believed that the continuous development of senior food using fruit is necessary.

According to data published by the National Institute of Agricultural Sciences in Korea, grapes were reported to be one of the fruits preferred by the elderly but difficult to consume, and the reason was found to be difficulty in peeling or cutting [18]. Grapes are a rich source of calcium, potassium, iron, vitamin E, and vitamin B, which can help relieve fatigue and increase vitality [19]. In addition, grapes contain flavonoids, which may prevent atherosclerosis and heart disease by inhibiting the formation of blood clots that block blood vessels. They are also rich in unsaturated fatty acids, which are effective in eliminating bad cholesterol. Additionally, grapes contain resveratrol, which has anti-cancer effects, and vitamins that help absorb calcium, making them a suitable fruit for the elderly to consume to prevent osteoporosis. In particular, the types of fruits that the elderly can eat are limited due to the nature of fruits, such as those with skins or tough, hard textures. Processing them into juice, jelly, puree, or mousse makes them easier to consume. Jelly, a food enjoyed from ancient to modern times, is made by mixing fruit and vegetable juice with a gelling agent and sugar, followed by concentrating and shaping [20]. Recently, puree has attracted attention as a food for the elderly because it is easy to chew and swallow [21]. Puree is made by crushing vegetables, fruits, etc., filtering them through a sieve, and making them into a liquid or paste that is easy to digest. If the raw materials contain functional properties, they can help alleviate or prevent chronic diseases, making puree a suitable option for the elderly of all genders and ages. Therefore, it is crucial to consider the value these products can add to meal options. A method to produce fruit jelly that is friendly to seniors has been studied by some researchers in Korea [22,23,24,25,26,27,28]. However, these studies were mainly conducted at the laboratory scale and did not investigate different fruits. It is important to note that food must be consumed with the tongue due to decreased chewing function in the elderly. In addition, few purees have been developed for the elderly, and pilot-scale research is insufficient for actual prototype production.

The aim of this study was to develop a fruit processed product for the elderly using grapes to supplement the dietary intake of the elderly and provide them with convenient food. To this end, we compared the yields at two production scales, lab-scale and pilot-scale, to investigate the feasibility of field application for the mass production of food for the elderly in the future. With these results, we hope to contribute to the development of food and diet for the elderly in preparation for an ultra-aging society and provide basic data for related companies and researchers.

## 2. Materials and Methods

### 2.1. Materials

The fruit sample used in this study was grape. Seedless *Kyoho* was used because older adults have reduced masticatory function and need to consume fruit without seeds. The grapes used in the experiment in this study were purchased from a local food market in Wanju, Jeollabuk-do. Supplementary materials were purchased from online shopping malls with sugar (CJ CheilJedang, Seoul, Republic of Korea), powdered agar (Woorigastory, Gyeonggi, Republic of Korea), xanthan-gum-based thickener (Toromi Perfect, DaesangWellife, Seoul, Republic of Korea), pectin (ESfood, Gyeonggi, Republic of Korea), and xanthan gum (ESfood, Gyeonggi, Republic of Korea).

### 2.2. Pre-Treatment of Grape

According to the Korean Industrial Standards for Seniors Friendly Foods (KS H 4897) for hardness and viscosity, it was first determined whether fresh fruit could be provided. For this purpose, we attempted to measure the physical properties according to the pre-treatment method for grapes, and the quality characteristics of grapes were analyzed according to the method in Section 2.4 (hardness, viscosity, soluble solids content, pH, titratable acidity and color). The pre-treatment methods were as follows: First, grapes were peeled and whole grapes were used as materials. Second, grapes were peeled, and were cut into 2, 4, and 8 pieces with a knife. Third, grapes were squeezed without their skins using a juicer (HE-DBF04, Hurom Co., Ltd., Seoul, Republic of Korea), made into grape juice, and filtered once through a sieve.

### 2.3. Preparation of Grape Jelly and Puree

Grape jelly and puree were prepared in three stages: stage 1 (able to eat with teeth), stage 2 (able to eat with gum), stage 3 (able to eat with tongue), according to the mastication stage. The products were divided into lab scale and pilot scale, and the formulas for grape products are presented in Table 1.

#### 2.3.1. Stage 1 (Able to Eat with Teeth)

**Figure 1:**
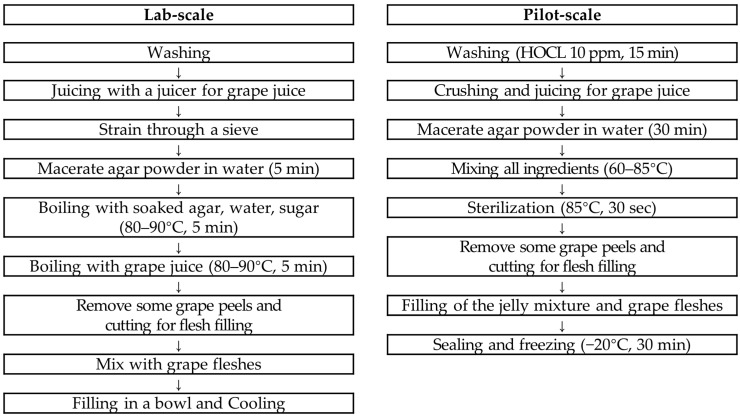
Recipes for the stage 1 grape jelly (able to eat with teeth).

The manufacturing process for grape jelly for the older adults able to eat with teeth is schematically presented (Figure 1). In the case of lab scale, grapes were removed from the branches and washed with water. Subsequently, grapes were placed in a juicer (HE-DBF04, Hurom Co., Ltd., Seoul, Republic of Korea) with their skins, squeezed, and filtered through a sieve. Next, the agar powder was put into water and soaked for 5 min, and then the soaked agar, water, and sugar were put into a pot and boiled at 80~90 °C for 5 min while stirring. After adding grape juice, the mixture was boiled for another 5 min while stirring to mix well with the agar. Meanwhile, the grapes to be filled in the grape jelly were peeled and cut into quarters with a knife to provide the texture of fresh grapes. The prepared grape flesh was added to the finished grape jelly, mixed well, placed in a container, cooled at room temperature for 30 min, and stored in the refrigerator.

In the pilot-scale case, the grapes were removed from the branches, soaked in 10 ppm HOCL for 15 min, and washed. Subsequently, grapes for grape juice production were crushed into small pieces with a crusher and squeezed with a pneumatic juicer. The agar powder was put into water and soaked for 30 min with stirring to prevent clumping. Next, grape juice and sugar were added to a heating stirrer and heated to 60 °C, and then mixed with the soaked agar and water. The temperature of the mixture was then raised to 85 °C, and then the mixture was heated for 30 s. The fleshes to be filled in jelly were prepared by peeling the skin and cutting into small pieces of about 1.5 cm or less. Small-cut grape fleshes were placed in the product container and filled with the heated mixture. Finally, the product container was sealed and cooled at −20 °C for 30 min, and then inspected for foreign matter using a metal detector, and stored in a refrigerator (0~10 °C).

#### 2.3.2. Stage 2 (Able to Eat with Gums)

**Figure 2:**
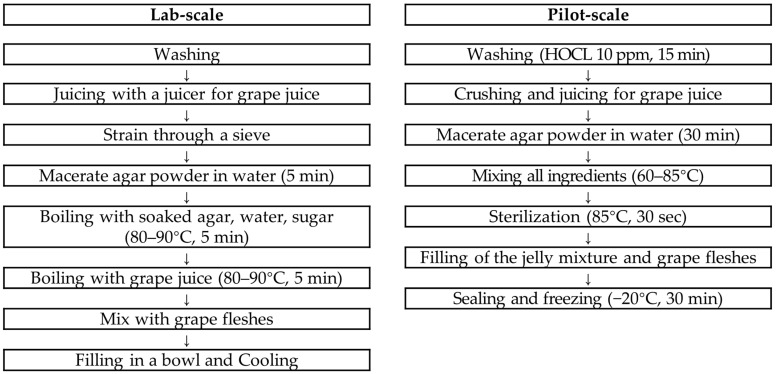
Recipes for the stage 2 grape jelly (able to eat with gums).

The manufacturing process for grape jelly for the older adults able to eat with gums is schematically presented (Figure 2). In the case of lab scale, grapes were washed in water, removed from the branches, placed in a juicer (HE-DBF04, Hurom Co., Ltd., Seoul, Republic of Korea) with their skins, extracted, and filtered through a sieve. Next, the agar was put into water and soaked for 5 min, and then the soaked agar, water, and sugar were put into a pot and boiled at 80~90 °C for 5 min while stirring. After adding grape juice, it was boiled for another 5 min while stirring to mix well with the agar. The finished grape jelly liquid was placed in a container, cooled at room temperature for 30 min, and stored in the refrigerator.

In the pilot-scale case, the grapes were removed from the branches, soaked in 10 ppm HOCL for 15 min, and washed. Grapes for grape juice production were crushed into small pieces with a crusher and squeezed with a pneumatic juicer. The agar was put into water and soaked for 30 min with stirring to prevent clumping. Grape juice and sugar were added to a heating stirrer and heated to 60 °C, and then mixed with the soaked agar and water. The temperature of the mixture was then raised to 85 °C, and the mixture was heated for 30 s. Finally, the product container was sealed and cooled at −20 °C for 30 min, and then inspected for foreign matter using a metal detector, and stored in a refrigerator (0~10 °C).

#### 2.3.3. Stage 3 (Able to Eat with Tongue)

**Figure 3:**
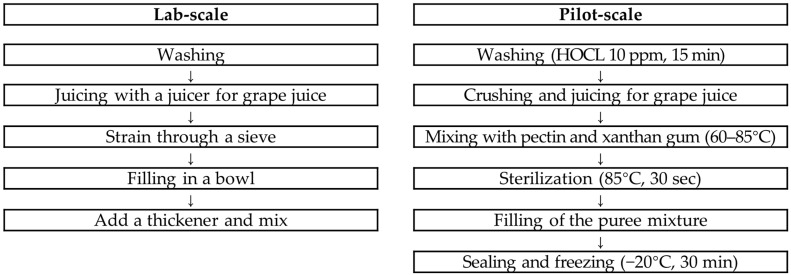
Recipes for the stage 3 grape puree (able to eat with tongue).

The manufacturing process for grape puree for the older adults able to eat with tongue was schematically presented (Figure 3). In the lab-scale case, the grapes were washed in water, removed from the branches, placed in a juicer (HE-DBF04, Hurom Co., Ltd., Seoul, Republic of Korea) with their skins, extracted, and filtered through a sieve. After the grape juice was placed in a container, a thickener was added and mixed. After 1 to 2 min, when the viscosity was formed, it was used as a sample.

In the pilot-scale case, the grapes were removed from the branches, washed by soaking in 10 ppm HOCL for 15 min, crushed into small pieces with a crusher, and extracted with a pneumatic juicer. A portion of the grape juice was taken and stirred to mix the pectin and xanthan gum well. After mixing the grape juice, pectin, and xanthan gum in a heated stirrer at 60 °C, the temperature of the mixture was raised to 85 °C, and the mixture was heated for 30 s. The product container was filled with the heated mixture, sealed, cooled at −20 °C for 30 min, inspected for foreign matter using a metal detector, and stored in a refrigerator (0~10 °C).

### 2.4. Comparison of Lab-Scale and Pilot-Scale Quality Characteristics of Grape Jelly and Puree

#### 2.4.1. Hardness and Viscosity

Hardness was measured using a texture analyzer (TA.XTplusC, Stable Micro Systems, Godalming, UK), according to the Korean Industrial Standards for Seniors Friendly Foods (KS H 4897) [29] and methods detailed by Kim et al. [28]. Stage 1 products were measured according to “experiment method 1” and “experiment method 3”. Stage 2 and 3 were measured according to “experiment method 2”.

The viscosity of stage 3 was measured using a rotational viscometer (DV-II+ Pro, Brookfield Engineering Laboratories Inc., Middleboro, MA, USA), according to the KS H 4897 [29]. After filling 500 mL of the sample into a glass beaker (600 mL, Ø90), a spindle (No. 62) was placed and measured at 12 rpm for 2 min.

#### 2.4.2. Fork and Spoon Test

In this study, a comparative analysis was conducted between the mastication stage according to the KS H 4897 and the International Dysphagia Diet Standardization Initiative (IDDSI) framework level [30]. The IDDSI test method (fork drip, spoon tilt, fork pressure test) was used to determine the level of the sample according to the IDDSI framework. The level of grape jelly was determined by the fork pressure test, a solid food classification method. This method was used at levels 6–7, and the level was determined by applying pressure with a fork until the thumb nail turns white. The level of grape puree was determined by the fork drip test and spoon tilt test, which are liquid classification methods. This method was used at levels 3–5, and the level of cohesiveness and adhesion was determined by placing liquid food on a fork or spoon and checking the falling pattern.

#### 2.4.3. Soluble Solids Content

The soluble solids content (SSC) was measured with a refractometer (PAL-1, Brix 0–53%, ATAGO, Tokyo, Japan). Samples were diluted 10-fold with distilled water and then homogenized for 1 min with a homogenizer (HG-15A, DAIHAN, Gangwon, Republic of Korea). The homogenized solution was centrifuged at 12,000 rpm for 5 min, and the supernatant of the separated sample was taken. The refractive index value obtained is expressed as percentage/degree Brix.

#### 2.4.4. pH and Titratable Acidity

The pH and titratable acidity (TTA) were measured using a pH-meter (Mettler Toledo S20K, Mettler-Toledo International Inc., Schwerzenbach, Switzerland). Each sample (4 g) was diluted 10-fold with distilled water and then homogenized for 1 min with a homogenizer (HG-15A, DAIHAN, Gangwon, Republic of Korea). The homogenized solution was centrifuged at 12,000 rpm for 5 min, and 30 mL of the supernatant was taken.

The titratable acidity, expressed as tartaric acid, was determined by titrating the supernatant with 0.1 N NaOH solution until the pH turned to 8.3. It was calculated according to the following equation:

$$\mathrm{Titratable}\;\mathrm{acidity}\left(\%\right)=\frac{V\times f\times 0.0075\times D}{S}\times 100$$

where V is the mL of 0.1 N-NaOH, f is the 0.1 N-NaOH Factor, D is the dilution factor, S is the sample amount (g).

#### 2.4.5. Color

The color of each sample was measured using a colorimeter (Ultra Scan PRO, Hunter Lab., Reston, VA, USA). Measurements were carried out by placing 9 g of samples in Petri dishes (40 mm). The results were expressed using CIE LAB parameters (L*; lightness, a*; (+) redness (−) greenness, b*; (+) yellowness, (−) blueness). The L*, a*, and b* values of the standard white plate used at that time were 99.57, −0.11, and −0.15, respectively. From these values, the color index (CI*) was obtained, according to the following equation:

$${CI}^{*}=(a^{*}\times 1000){/(L}^{*}\times b^{*})$$

### 2.5. Statistical Analysis

All experiments, except hardness measurement, were performed in triplicate and expressed as mean and standard deviation. Data analysis was performed with SPSS 27.0 (IBM SPSS Statistics, IBM, Armonk, NY, USA). Statistical significance of comparisons between the two scales was performed by the independent *t*-test, and the level of significance was set at α = 0.05. To visually represent the association among physicochemical properties of grape samples (jelly and puree), a heatmap based on Pearson correlation coefficient was presented. The correlation heatmap was generated using the R studio (R version 4.3.3).

## 3. Results and Discussion

### 3.1. Quality Characteristics of Grapes According to the Pre-Treatment Methods

The results of the analysis of the soluble solids content (SSC), pH, titratable acidity (TTA), and color of the grapes used for the production of grape processed products for the lab-scale and pilot-scale are presented in Table 2. The analysis sample was obtained by crushing the grapes with their skins and making grape juice. The sugar content (SSC) of the grapes used in this study was 20.00 °Brix. This value is consistent with a previous study [31] that reported the highest-quality Kyoho grapes’ sugar content to be over 18.00 °Brix, thereby confirming that the sugar content of the grapes utilized in this study was appropriate. Meanwhile, grapes have a higher sugar content than bokbunja (7.60 °Brix), strawberries (13.18 °Brix), and mulberries (11.00 °Brix) [32,33,34]. This suggests that grape raw material may be a preferable choice for reducing the amount of sugar in the production of processed products, such as jelly. The pH and TTA of grapes are 3.90 and 0.65%, respectively. According to a study by Park et al. [31], the acidity of grapes (Kyoho) was reported to be 0.4~0.6%. In this study, the TTA of the grapes was found to be 0.65%, showing similar results. The sugar–acid ratio is a crucial factor when consumers purchase fruit or fruit juice, and is the most commonly used indicator for the quality evaluation of processed fruit products [35]. The sugar–acid ratio of the grapes used in this study was found to be 31.16, and the color analysis showed that the L* value was 43.12, the a* value was 35.99, the b* value was 3.97, and the CI* value was 210.88, indicating a bright purple color.

The results of the hardness and viscosity measurements results of the grapes according to the pre-treatment methods are presented in Table 3. The hardness values of the peeled grape flesh and grapes cut into two equal parts were 73,564.95 N/m^2^ and 57,531.56 N/m^2^, respectively. These values correspond to stage 1 of the senior-friendly food standard of Korea (KS H 4897), that is, can be eaten with teeth. The hardness values of the grapes cut into four and eight pieces were 13,475.12 N/m^2^ and 10,849.06 N/m^2^, respectively. It was confirmed that both pre-treatment methods comply with KS H 4897 stage 3, i.e., can be eaten with tongue. When squeezed in the form of grape juice, the hardness was 311.94 N/m^2^, which met the stage 3 hardness standards for KS H 4897. However, the viscosity was 135.50 mPa·s, which did not meet the viscosity standard. As a result, it was found that depending on the pretreatment method, the grapes corresponded to the first and third stages of senior-friendly food. However, the consumption of grapes in pre-treatment form is not suitable for the elderly due to the risk of aspiration into the respiratory tract [36].

Based on these results, the present study aimed to process fresh grapes into a safer form rather than using them in their raw form. Based on the results and in consultation with experts from aging research organizations, medical institutions, and academia, two processed products were selected: jelly and puree.

### 3.2. Comparison of Lab-Scale and Pilot-Scale Quality Characteristics of Grape Jelly and Puree

#### 3.2.1. Hardness and Viscosity

The results for the hardness and viscosity of the grape jelly and puree are presented in Table 4. The physical properties of food are among the important factors that can satisfy the preferences and demands of consumers. In this study, jelly was selected as the form for both stage 1 and 2. The hardness results were then compared based on the production scale. When the production scale was changed from lab scale to pilot scale in the stage 1 jellies, the hardness value significantly increased from 191,551.82 N/m^2^ to 236,445.30 N/m^2^ (*p* < 0.05) in the juice portion, and significantly increased from 127,654.05 N/m^2^ to 225,127.61 N/m^2^ (*p* < 0.05) in the flesh portion. In the stage 2 jellies, when the scale was changed from lab to pilot, the hardness value showed a significant decrease from 44,266.29 N/m^2^ to 25,302.45 N/m^2^ (*p* < 0.05).

The product in stage 3 was manufactured into puree. The results showed a significant decrease in hardness from the lab scale (366.06 N/m^2^) to the pilot scale (315.13 N/m^2^) (*p* < 0.05), and a decrease in viscosity from 2495.93 mPa∙s to 1561.33 mPa∙s (*p* < 0.05). The viscosity of foods varies depending on factors such as temperature, solids content, and the type of thickener used [37]. To modify the viscosity of liquid, commercial thickeners (CT) are commonly used in most nursing facilities in Korea [38]. Furthermore, most of these facilities use xanthan-gum-based thickening agents and mixed agents as CT agents. In this study, lab-scale puree was manufactured using xanthan-gum-based thickeners, which are also commonly used in commercial nursing facilities. Consequently, it appears to have higher hardness and viscosity than the pilot-scale puree made with pectin and xanthan gum.

#### 3.2.2. Fork and Spoon Test

In 2016, the International Dysphagia Diet Standardisation Initiative (IDDSI) developed the IDDSI framework, to describe texture-modified foods and thickened liquids used for individuals with dysphagia of all ages, of all cultures, and in all care settings [30]. In Korea, food texture is classified into three stages based on chewing ability, while the internationally recognized IDDSI level is subdivided into eight levels. However, it is possible that this may be challenging to implement directly in real-world settings, such as nursing facilities. Therefore, in identifying the IDDSI level that meets our country’s industrialization standards, it is necessary for nursing facilities and the older adults at home to select foods that are suitable for their ability to chew easily without using a hardness tester or viscometer. The results of the IDDSI standard classification for grape jelly and puree are presented in Table 5. The jelly was classified according to the IDDSI level using the fork pressure test at both mastication levels. After analyzing both the lab-scale and pilot-scale results, it was determined that the stage 1 jelly is classifiable as level 7 (easy to chew), while the stage 2 jelly was classified as level 6 (soft and bite-sized). Level 7 is the point at which regular food can be consumed, meaning that the food can be easily broken apart with the side of a fork or spoon, and when pressed with the tines of a fork, the thumb nail blanches to white. The stage 1 jelly is a food for older adults that can be consumed using teeth. It was manufactured into a harder jelly by increasing the agar content and was level 7 according to IDDSI standards. Additionally, we added grape fleshes to the jelly to make it easier for the older individuals to consume raw fruit and to fulfill their desire to chew. Level 6 foods are those with a soft texture and can be crushed or broken with a fork or spoon. For instance, jelly at level 6 has less agar and no grape flesh, which makes it softer and more brittle than level 7 jelly. When pressed with a fork, it breaks into smaller pieces and does not return to its original shape.

The IDDSI-level classification of the grape puree was determined to be at stage 3 by using two distinct methods: the fork drip test and the spoon tilt test methods. When the puree was scooped up with a fork and spoon and tilted, it flowed slowly from the fork prongs and spoon, forming long prongs. The puree did not flow immediately as it fell, but gradually accumulated and scattered over the bowl, and when the fork and spoon were held flat, some of the puree formed dollops at the bottom. This appearance confirmed that both the lab scale and the pilot scale corresponded to IDDSI level 3 (moderately thick). IDDSI level 3 is an intermediate level that applies to both foods and drinks, and indicates a food with a viscosity similar to “honey density” [30]. For the grape puree in this study, it was judged that it cannot be consumed with a fork because it falls slowly through the prongs of a fork, and that it should be consumed by scooping up with a spoon.

The commercially available thickeners used in lab-scale purees should be added to the food according to the manufacturer’s recommendations and consumed immediately after viscosity is formed 1–2 min later. However, in real-world settings, such as assisted living and nursing facilities, there may be cases in which the appropriate intake time is not met. In addition, the rheology of the viscosity enhancer varies depending on the type of product, concentration, temperature, and time [39,40]. It is reported that the viscosity of food using viscosity enhancers decreases with increasing temperature [41]. Therefore, since it is costly to put the same commercial thickener in the pilot-scale product as in the lab-scale, and there is a difference in viscosity over time, we replaced it with an ingredient contained in the commercial thickener (a mixture of pectin and xanthan gum) to develop the puree.

#### 3.2.3. Soluble Solids Content

The soluble solids content (SSC) of the grape jelly and puree and the comparison results between the lab scale and pilot scale are presented in Table 6. The SSC of the stage 1 jelly was 22.33 °Brix at the lab scale and 18.67 °Brix at the pilot scale, and the SSCs of the stage 2 jelly were 23.00 °Brix and 18.33 °Brix, respectively. The stage 3 puree was 20.00 °Brix at the lab scale and 18.90 °Brix at the pilot scale. The SSC showed a significant difference between the lab scale and pilot scale at all stages (*p* < 0.05), and as the production scale increased to pilot scale, the SSC decreased. This is believed to be the result of the differences in the process, depending on the production scale. In particular, the SSC of jelly for lab-scale use was high at 20.00~23.00 °Brix. Agar, an ingredient of jelly, is made of dietary fiber and has a high water absorption capacity [42,43]. In this study, agar water and sugar were mixed and heated to promote the elution of the SSC on a lab scale. In addition, it is believed that the sugar content was high because there was little destruction of the SSC component in the grapes due to the short production time. According to a previous study [44], the SSC of Sansuyu puree-added jelly was 64.31 to 66.96 °Brix, showing different results from the grape jelly and puree in this study. This is thought to be due to differences in the SSC of the final product, depending on the characteristics of the fruit and the mixing ratio. Based on the analysis, it is recommended to produce products that take into account quality characteristics, such as SSC. These characteristics can vary, even when the same fruit is harvested and stored under similar conditions.

#### 3.2.4. pH

The pH results for the grape jelly and puree are presented in Table 6. As a result of the pH analysis, the stage 1 jelly was found to be 4.04 and 4.14 on the lab scale and pilot scale, respectively, and the stage 2 jelly was found to be 4.03 and 4.10. Additionally, in the case of the stage 3 puree, the two scales were 3.90 and 3.82, respectively. The pH showed a significant difference between the lab scale and pilot scale at all stages (*p* < 0.05). In a study on the quality characteristics of jelly according to the type of gelling agent, it was reported that gelatin had a slightly acidic characteristic, with a pH of 6.38, and carrageenan and konjac had a near-neutral characteristic, with pH 7.03 and 7.01, respectively [45]. Therefore, even for the same fruit, physical properties such as pH are expected to change significantly, depending on the gelling agents used together, and it seems necessary to select an appropriate gelling agent according to the characteristics of the fruit.

#### 3.2.5. Titrable Acidity

The results of the titratable acidity (TTA) analysis of the grape jelly and puree are presented in Table 6. The TTA of the stage 1 jelly was 0.42% at the lab scale and 0.39% at the pilot scale (*p* < 0.05). In the case of the stage 2 jelly, the TTA value was 0.46% at the lab scale and the 0.43% at the pilot scale. The stage 3 puree was 0.68% at the lab scale and 0.74% at the pilot scale (*p* < 0.05).

The sugar–acid ratio is one of the most important factors in consumer evaluation of sensory quality, and can be used as an efficient indicator to predict consumer preference [46,47]. As a result of analyzing the sugar–acid ratio for grape jelly and grape puree according to the SSC and TTA results, the sugar–acid ratio values of the stage 1 jelly were 52.76 and 48.40 on the two scales, respectively.

In the case of the stage 2 jelly, the lab-scale and pilot-scale values were 50.11 and 42.44, respectively. In addition, the stage 3 puree values were 29.57 and 25.80, respectively. The sugar–acid ratio showed a significant difference between the lab scale and pilot scale at all stages (*p* < 0.05), and as the production scale increased to pilot scale, the sugar–acid ratio decreased. According to previous studies, the sugar–acid ratio of fruit juice mixed with red beet and apple juice was reported to be 13.92 to 27.06 [48], and the sugar–acid ratio of citrus juice was reported to be 15 to 18 [49]. The grape jelly manufactured in this study shows slightly higher values compared to previous studies, which is believed to be due to the difference in sugar content and acidity of the raw fruit and the presence or absence of added sugar.

#### 3.2.6. Color

Color is an important quality indicator for consumers that influences product preference and purchase decisions [50]. In this study, we analyzed the color difference between grape jelly and puree, according to the manufacturing process, between lab scale and pilot scale (Table 6). The L* value (lightness) of the jelly significantly decreased with increasing production scale at the pilot scale for both jelly products (stage 1: from 34.69 to 28.16, stage 2: from 32.86 to 26.03) (*p* < 0.05). The a* value (redness) was found to decrease with scale change, similarly to the L* value (stage 1: from 24.57 to 17.54, stage 2: from 26.78 to 17.53) (*p* < 0.05). In the case of the b* value (yellowness), the jelly at stage 1 increased from 3.97 to 5.07 and that at stage 2 from 4.95 to 4.96. In the case of the stage 3 puree, the L* value and the a* value decreased as the scale of the process increased from lab scale to pilot scale (L*: from 44.78 to 27.20, a*: from 21.76 to 17.68) (*p* < 0.05). On the other hand, the b* value increased from 3.33 to 5.57 (*p* < 0.05).

It appears that grapes are a representative fruit containing a large amount of anthocyanin [51], and it has been reported that the stability of anthocyanin pigments contained in grapes is affected by conditions such as temperature. According to a previous study [52], the a* value (redness) of the pigment in purple sweet potatoes decreased rapidly as the temperature increased from 60 °C to 80 °C. In addition, in a study [53] in which the anthocyanin content of aronia was changed, the anthocyanin content decreased rapidly above 60 °C, and at 70 °C, the total anthocyanin content was halved in 24 h. In this study, as the scale of production increased from lab scale to pilot scale, it seems that the anthocyanin content of grape juice was affected by temperature changes during heating and the addition of the sterilization process. Accordingly, it is believed that the color of the processed grape product changed from light purple to dark brown.

Meanwhile, the color index (CI*) values calculated from these color data are presented in Table 6. The CI* appeared different as it increased from the lab scale to the pilot scale, with the stage 1 jelly going from 179.06 to 122.99 (*p* < 0.05), and the stage 2 jelly going from 164.76 to 135.84 (*p* < 0.05). In addition, the CI* of the stage 3 puree was 145.81 for the lab scale and 117.05 for the pilot scale (*p* < 0.05). The CI* value indicates the intensity of the color, such as fading or darkening. This attribute is important in evaluating the quality, freshness, and marketability of fruit and serves as a visual consumer indicator that influences marketing and quality control [50]. In this study, it was found that due to differences in production, the CI* value of the pilot-scale product was lower and had a darker and deeper color compared to the lab-scale product. These results seem to be due to differences in heating time and temperature, and it is believed that an accurate targeting method tailored to each consumer through surveys of consumer preferences for the product is needed.

### 3.3. Correlation Heatmap

**Figure 4:**
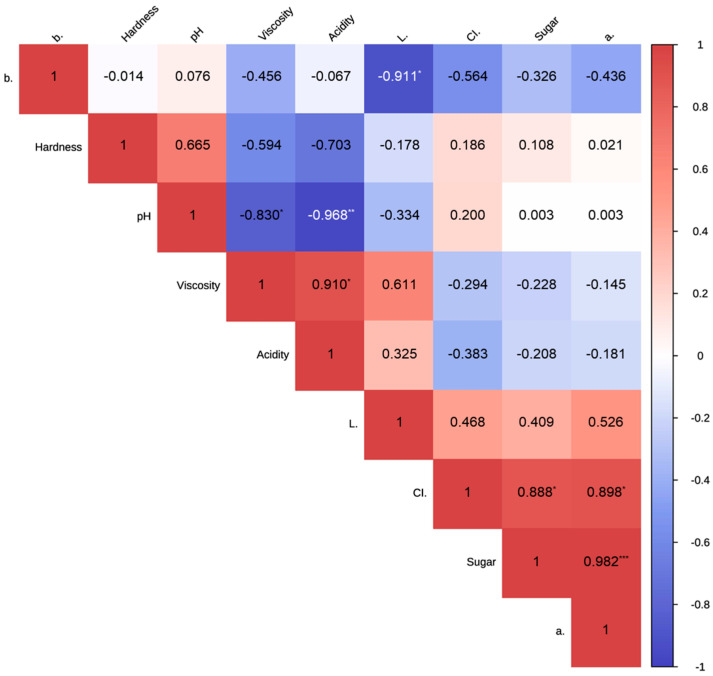
Correlation heatmap of physicochemical properties of grape jelly and puree. * *p* < 0.05; ** *p* < 0.01; *** *p* < 0.001.

To investigate the correlations among the physicochemical properties of the grape samples (jelly, puree), a Pearson correlation analysis was performed, and the results were visualized in the form of a heatmap (Figure 4). The variables that showed significant positive correlations between physicochemical properties were acidity and viscosity (r = 0.910, *p* < 0.05), a* and sugar content (r = 0.982, *p* < 0.001), CI* and sugar content (r = 0.888, *p* < 0.05), and a* and CI* (r = 0.898, *p* < 0.05). Conversely, significant negative correlations were found between pH and viscosity (r = −0.830, *p* < 0.05), pH and acidity (r = −0.968, *p* < 0.01), and b* and L* (r = −0.911, *p* < 0.05).

## 4. Conclusions

This study was conducted to develop a grape (*Kyoho*) product that can facilitate food intake for elderly people with chewing difficulties. In addition, the possibility of field application for future prototype production was investigated by comparing lab-scale and pilot-scale production. The stages (stage 1: able to eat with teeth, stage 2: able to eat with gums, stage 3: able to eat with tongue) of the products were determined according to KS H 4897, and the physicochemical properties were measured according to the general test method of the Food Code. As a result of this study, when the production scale was changed from lab scale to pilot scale in stage 1, the hardness increased significantly. The hardness in stage 2 and 3 and the viscosity in stage 3 also showed a significant decrease. The pH and sugar–acid ratio showed a significant difference between the two scales at all stages. The results of this study confirmed the possibility of developing customized grape products for the elderly who have difficulty chewing, and the importance of maintaining product quality during scaled-up production was confirmed through the differences in physicochemical properties between lab-scale and pilot-scale production. It is thought that these results can provide important information for future industrialization and field applicability.

## Figures and Tables

**Table 1:** Formulas for grape jelly and puree.

Ingredients	Lab-Scale		Pilot-Scale	
	g	%	kg	%
Stage 1 (able to eat with teeth)				
Grape Juice	250.0	47.2	23.6	47.2
Grape flesh	156.0	29.4	14.7	29.4
Agar powder	7.0	1.3	0.7	1.3
Sugar	28.5	5.4	2.7	5.4
Water	88.5	16.7	8.4	16.7
Total	530.0	100.0	50.1	100.0
Stage 2 (able to eat with gum)				
Grape Juice	250.0	68.8	34.4	68.8
Agar powder	3.6	1.0	0.5	1.0
Sugar	27.0	7.4	3.7	7.4
Water	83.0	22.8	11.4	22.8
Total	363.6	100.0	50.0	100.0
Stage 3 (able to eat with tongue)				
Grape Juice	200.0	98.0	48.5	96.9
Thickener ^(1)^	4.0	2.0	-	-
Pectin	-	-	1.5	3.0
Xanthan gum	-	-	0.05	0.1
Total	204.0	100.0	50.1	100.0

^(1)^ For the lab-scale puree, a commercially available thickener (Daesang Wellife, Seoul, Republic of Korea) was used.

**Table 2:** Soluble solids content (SSC), pH, titratable acidity (TTA), and color of grapes.

Properties	SSC (°Brix)	pH	TTA (%)	Sugar–Acid Ratio	Color			
					L*	a*	b*	CI*
Grape (*Kyoho*)	20.00 ^(1)^ ± 1.00	3.90 ± 0.03	0.65 ± 0.10	31.16 ± 3.23	43.12 ± 0.32	35.99 ± 0.43	3.97 ± 0.31	210.88 ± 16.05

^(1)^ Mean (*n* = 3) ± standard deviation.

**Table 3:** Hardness and viscosity of grapes according to the pre-treatment methods.

Properties	Hardness (N/m^2^)		Viscosity (mPa·s)	KS Stage ^(2)^
	Method ^(1)^	Mean ± S.D.	Mean ± S.D.	
Raw fruits	3	73,564.95 ± 1414.71	-	1
	1	68,075.87 ± 683.29		
Cut into 2 pieces	3	57,531.56 ± 2933.61	-	1
	1	42,441.32 ± 1413.24		
Cut into 4 pieces	3	28,294.21 ± 1844.56	-	3
	1	25,804.32 ± 2561.71		
	2	13,475.12 ± 627.97		
Cut into 8 pieces	3	22,163.80 ± 2098.35	-	3
	1	20,541.60 ± 1992.12		
	2	10,849.06 ± 1298.28		
Juicing	2	311.94 ± 19.80	135.00 ± 2.50	none ^(3)^

^(1)^ In cases in which stages 2 and 3 were obtained by testing according to the 3 methods of KS H 4897, the final stage was confirmed by retesting according to method 2. ^(2)^ Korean Industrial Standards, KS H 4897 (stage 1: 500,000~50,000 N/m^2^, stage 2: 50,000~20,000 N/m^2^, stage 3: ~20,000 N/m^2^, over 1500 mPa·s). ^(3)^ Stage determination is not possible due to non-compliance with viscosity standard of KS H 4897 (over 1500 mPa·s).

**Table 4:** Hardness and viscosity of grape jelly and puree.

Properties	Method ^(1)^	Part	Scale	Hardness (N/m^2^)		Viscosity (mPa·s)	
				Mean ± S.D.	*p*-Value ^(2)^	Mean ± S.D.	*p*-Value ^(2)^
Stage 1, Jelly (able to eat with teeth)	3	Juice	Lab	191,551.82 ± 4710.58	0.005	-	-
			Pilot	236,445.30 ± 21,012.05		-	
		Flesh	Lab	127,654.05 ± 10,607.22	<0.001	-	-
			Pilot	225,127.61 ± 65,082.81		-	
	1	Juice	Lab	114,235.05 ± 5722.79	0.131	-	-
			Pilot	166,454.85 ± 37,577.77		-	
		Flesh	Lab	88,260.97 ± 7879.01	0.415	-	-
			Pilot	93,319.97 ± 32,818.96		-	
Stage 2, Jelly (able to eat with gums)	2		Lab	44,266.29 ± 1303.71	<0.001	-	-
			Pilot	25,302.45 ± 1290.15		-	
Stage 3, Puree (able to eat with tongue)	2		Lab	366.06 ± 10.65	<0.001	2495.93 ± 85.65	<0.001
			Pilot	315.13 ± 0.00		1561.33 ± 25.17	

^(1)^ According to the test method of KS H 4897, stage 1 was tested using methods 1 and 3, and stages 2 and 3 were tested using method 2. ^(2)^ *p*-value by *t*-test.

**Table 5:** IDDSI framework levels of grape jelly and puree, according to the IDDSI testing methods.

Scale Type	Cooking Method	Picture	KS Stage ^(1)^	IDDSI ^(2)^		Expression of Texture			Description
				Level ^(3)^	Test				
Lab	Jelly	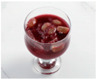	1	7 (EC)	Fork Pressure	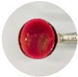	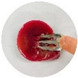	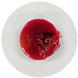	It is completely squashed and does not regain its shape.
	Jelly	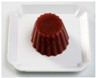	2	6 (SB)	Fork Pressure	^ 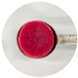 ^	^ 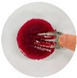 ^	^ 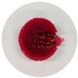 ^	It can be broken apart into smaller pieces to press with a fork.
	Puree	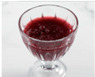	3	3 (MO)	Spoon Tilt, Fork Drip	^ 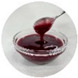 ^	^ 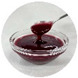 ^	^ 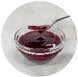 ^	Drips slowly in dollops through the prongs of a fork.
Pilot	Jelly	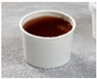	1	7 (EC)	Fork Pressure	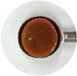	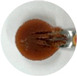	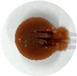	It is completely squashed and does not regain its shape.
	Jelly	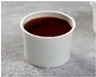	2	6 (SB)	Fork Pressure	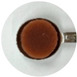	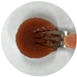	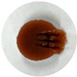	It can be broken apart into smaller pieces to press with a fork.
	Puree	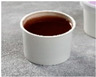	3	3 (MO)	Spoon Tilt, Fork Drip	^ 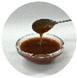 ^	^ 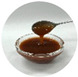 ^	^ 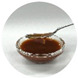 ^	Drips slowly in dollops through the prongs of a fork.

^(1)^ Korean Industrial Standards for Seniors Friendly Foods (KS H 4897: 2022) [29]. ^(2)^ International Dysphagia Diet Standardisation Initiative (IDDSI: 2019) [30]. ^(3)^ Level 7, EC: easy to chew; Level 6, SB: soft and bite-sized; Level 3, MO: moderately thick.

**Table 6:** Soluble solids content (SSC), titratable acidity (TTA), pH, and color of grape jelly and puree.

Properties	Stage 1, Jelly (Able to Eat with Teeth)			Stage 2, Jelly (Able to Eat with Gum)			Stage 3, Puree (Able to Eat with Tongue)		
	Lab	Pilot	*p*-Value ^(1)^	Lab	Pilot	*p*-Value	Lab	Pilot	*p*-Value
SSC (°Brix)	22.33 ± 0.58 ^(2)^	18.67 ± 0.58	0.001	23.00 ± 0.01	18.33 ± 0.58	0.005	20.00 ± 0.01	18.90 ± 0.01	<0.001
pH	4.04 ± 0.01	4.14 ± 0.02	0.001	4.03 ± 0.01	4.10 ± 0.01	0.002	3.90 ± 0.01	3.82 ± 0.01	<0.001
TTA (%)	0.42 ± 0.01	0.39 ± 0.01	0.018	0.46 ± 0.01	0.43 ± 0.02	0.116	0.68 ± 0.02	0.74 ± 0.01	0.004
Sugar–acid ratio	52.76 ± 0.50	48.40 ± 2.23	0.030	50.11 ± 1.23	42.44 ± 2.11	0.006	29.57 ± 0.67	25.80 ± 0.44	0.001
Color value									
L*	34.69 ± 0.56	28.16 ± 0.52	<0.001	32.86 ± 0.62	26.03 ± 0.08	0.002	44.78 ± 0.03	27.20 ± 0.24	<0.001
a*	24.57 ± 0.25	17.54 ± 0.01	<0.001	26.78 ± 0.43	17.53 ± 0.24	<0.001	21.76 ± 0.11	17.68 ± 0.29	<0.001
b*	3.97 ± 0.30	5.07 ± 0.09	0.004	4.95 ± 0.07	4.96 ± 0.25	0.920	3.33 ± 0.05	5.57 ± 0.41	0.010
CI*	179.06 ± 16.33	122.99 ± 3.84	0.004	164.76 ± 2.63	135.84 ± 5.69	0.001	145.81 ± 2.70	117.05 ± 6.86	0.002

^(1)^ *p*-value by *t*-test, ^(2)^ Mean (*n* = 3) ± standard deviation.

## Data Availability

The data from this study are available in the article.
